# South African physiotherapists’ perspectives on the competencies needed to work in special schools for learners with special needs

**DOI:** 10.4102/sajp.v77i1.1571

**Published:** 2021-11-23

**Authors:** Machuene C. Manamela, Carina A. Eksteen, Bhekiwe Mtshali, Shade A.S. Olorunju

**Affiliations:** 1 Department of Physiotherapy, Faculty of Health Care Sciences, Sefako Makgatho Health Sciences University, Pretoria, South Africa; 2 Biostatistics Unit, South African Medical Research Council, Pretoria, South Africa

**Keywords:** competencies, framework, special needs, special schools, policies and procedures

## Abstract

**Background:**

Investigation into, and description of competencies in the various sectors in which the physiotherapy profession is practised, contribute to the standardisation of practice, professional education, and guides research and administration, and is necessary in South Africa.

**Objective:**

To identify the competencies implemented by physiotherapists working in an educational setting for learners with special needs and to determine physiotherapists’ opinions on the identified competencies.

**Methods:**

A sequential mixed method research design was implemented to explore the competencies that physiotherapists implement during their intervention for children with special needs through focus group discussions (FGDs). A questionnaire based on the statements that emerged from the thematic analysis of the transcribed FGDs, and validated, was implemented in a cross-sectional survey amongst all physiotherapists employed in special schools. SPSS version 24 was used for the analysis of closed responses and thematic analysis was done on open-ended responses (*n* = 22).

**Results:**

The respondents’ knowledge and skills regarding physiotherapy theories and implementation ranged from ‘good’ to ‘very good’. However, integration of the therapeutic knowledge and skills in different aspects of the special educational environment, and community integration, were rated ‘poor’ to ‘fair’. Support of physiotherapists to implement policies and procedures, and to attend continuing professional development, ranged from ‘fair’ to ‘poor’.

**Conclusion:**

Lack of knowledge in educational policies and procedures in classroom strategies negatively influence the integration of therapeutic strategies in the special educational environment.

**Clinical implications:**

The contribution of our study to learners with special needs in schools was outlined.

## Introduction

Competency frameworks are developed globally to describe the essential foundational competencies in all aspects of a healthcare profession, to guide practising the profession, serve as the directive and foundation for the education of healthcare professionals and to reveal areas in which research is lacking. A framework is defined as a set of principles and ideas that you use when you are forming your decisions and judgments (Macmillan Dictionary, Macmillan Publishers Limited 2007).

The foundational competencies for the physiotherapy profession have been developed generically, as well as for community-based education (Mostert-Wentzel, Frantz & Van Rooijen 2013) as one of the aspects of physiotherapy practice. Although the generic competencies of physiotherapy are the same for the different healthcare sectors that physiotherapists are working in, they are differently applied in various healthcare sectors, because of the unique characteristics and demands in each sector. On an international level, the World Confederation of Physical Therapy (WCPT 2019) acknowledged the fact that the practice of the profession varies globally depending on the healthcare system and facilities within which physiotherapy is being practised. The application of the competencies are influenced by each country’s laws, regulations, human and monetary resources, which require contextualisation of foundational competencies. In the educational setting for learners with special needs, the purpose of physiotherapy may demand a different variety and/or combination of competencies from a physiotherapist than what is required from a physiotherapist, who for instance, works in the community. Although physiotherapists globally, may have acquired similar competence to work in comparable environments, contextualisation of these competencies in the respective work settings is essential (WCPT 2019).

During the past decade, the South African Society of Physiotherapy (SASP) encouraged physiotherapists to develop evidence-based statements, competency frameworks and/or clinical practice guidelines for the management of certain conditions, to promote standardised physiotherapy practice throughout the country. In line with this recommendation, Pillay (2011) recommended that the SASP, in conjunction with the Department of Basic Education (DoBE), draw up clinical practice guidelines for physiotherapists working in educational settings, similar to what has been done in the United States of America (USA) and Australia. Our current study aimed to take the initial steps to achieve this goal.

The development of a competency framework for physiotherapy management of learners with special needs should not only be benchmarked with international competency frameworks, but should, most importantly, also be relevant to the South African context.

Although it can be assumed that the generic competencies for physiotherapy are also relevant for physiotherapy practice in an educational setting, the specific competency framework for physiotherapists in educational settings, such as schools for learners with special needs, will enrich the clinical practice, and physiotherapy education across South Africa (SA).

The purpose of our study was to develop a contextualised competency framework that will inform physiotherapy practice in schools for learners with special needs. The preparatory process in the development of a contextualised competency framework entailed the exploration of the actions and experiences of the physiotherapists working in special schools for learners with special needs within an inclusive educational policy in SA. In this article, the authors reports on the explored perspectives of South African physiotherapists on their activities and experiences in schools for learners with special needs.

## Methods

A sequential mixed method research design was used to explore the opinions on the perceived competencies of physiotherapists employed in schools for learners with special needs in South Africa. Focus group discussions (FGDs) were held with the physiotherapists employed in special schools, in one province and the thematic analysis of the transcribed FGDs, resulted in a categorised list of statements that reflected the respondents’ competencies. Based on the list of competencies, we compiled a questionnaire which was contextualised with the statements of the competency framework compiled by Effgen, Chiarello and Milbourne (2007). The questionnaire was used to conduct a cross-sectional survey amongst the physiotherapists employed at schools for learners with special needs throughout the country (excluding the physiotherapists in the province where the FGDs were held). Permission to contextualise the list of competence statements with the competency framework by Effgen et al. (2007) was obtained from the first author before the questionnaire was finalised.

The questions were formulated in the idiomatic language and context, familiar to SA physiotherapists. For example, the questions that required knowledge of the Canadian legislation governing physiotherapists in Canada (such as the *Individuals with Disabilities Education Act* [IDEA]), were replaced with relevant knowledge questions on SA policy (*Education White Paper Six*).

The respondents had to answer each question on a 5-point Likert scale (ranging from very poor to very good). The questions were categorised in the nine competency areas similar to the categories described by Effgen et al. (2007) namely, the context of physiotherapy practice in schools, wellness and prevention activities by physiotherapists in schools, team collaboration, examination and evaluation of children, planning, intervention, documentation, administrative issues handled by physiotherapists in schools and research.

### Pilot study

The questionnaire was piloted on the physiotherapists who participated in the FGDs prior to administering the cross-sectional survey on physiotherapists in the rest of the country. The purpose of the pilot study was to refine the formulation of the questions for content, clarity and length of questions, and to check if a preliminary analysis of the answers on the questionnaire would answer the research question. The results of the preliminary analysis were not incorporated in the main study because the participants in the pilot study were part of the FGDs in the previous phase.

No changes in the formulation of the questions were suggested by the participants. The analysis indicated that the outcome of the questionnaire would answer the research question. The eligibility of the questionnaire was objectively determined by performing Cronbach’s Alpha. The Cronbach’s Alpha for the 26 items in the questionnaire was 0.755 (unstandardised) and the standard coefficient was found to be 0.932. The questionnaire’s results showed that it had internal consistency and was a reliable instrument to use in phase 2 of our study (Kaiser 1974).

### Sampling procedure

At the commencement of data collection, it was found that only 48 of the 58 potential participants that were recruited, met the inclusion criteria.

We emailed the consent form and more details on the aims and objectives, as well as the questionnaire, to all the physiotherapists who agreed to participate in our study. The participants were requested to complete the questionnaire, sign the consent form and email them back to the team.

### Data analysis of the questionnaire

The responses on each questionnaire were entered into SPSS version 24. One of our team captured the raw data and it was checked by a colleague prior to the calculations. A statistician performed the data analysis. Descriptive statistics were used to analyse the data presenting summary statistics. Furthermore, analyses of cross-tabulation and correlation were undertaken to evaluate the degree of association and correlation between knowledge questions and skill characteristics.

### Ethical considerations

Ethical approval to conduct the study was obtained from the former Medical University of Southern Africa (MEDUNSA) Research Ethics Committee (reference number: MREC/H/96/2014). Consent to send the questionnaire to all physiotherapists employed at schools for children with special needs, were obtained from only three of the eight provincial coordinators in the National Department of Basic Education (DoBE). Subsequent to this inadequate response, the first author continued to obtain permission to conduct the survey from the directors and heads of the departments of Basic Education in the five provinces that employed physiotherapists in schools. In addition, consent was obtained from the principals of the schools who employed physiotherapists. Physiotherapists employed in the schools were telephonically informed about the aims, objectives and procedure of our study and asked whether they would voluntarily participate. Data were stored in a safe password protected computer.

## Results

Three provinces did not have physiotherapists employed in special schools. Only 22 (45.8% of the total population) participants returned the completed questionnaire and signed consent forms. Each completed questionnaire was assigned a code. At the time when data were collected, only five of the eight (excluding Gauteng) had physiotherapists in special schools. The response rate per province is shown in Table 1.

**TABLE 1:** Response rate per province.

Province	No. of special schools	Total no. of physiotherapists	No. of physiotherapists participated	Response rate (%)
Province 1	1	6	4	66.7
Province 4	2	5	3	60.0
Province 2	5	14	8	57.1
Province 5	1	2	1	50.0
Province 3	5	21	6	28.6

**Total**	**14**	**48**	**22**	**45.8**

### Demographic information of study respondents

Of the 22 physiotherapists who participated, the majority 20 were females, and the minority 2 were males. This female: male ratio was similar to the Australian study by Schofield and Fletcher (2007) who found that physiotherapy remains a female dominated profession although the proportion of male physiotherapists is increasing.

The age ranged from 28 to 65 years with a mean of 42.4 ± 11.4 years. A greater proportion (7) were in the age group 30–39 years followed by those in the age group 50–59 years (6). There were four respondents in the age group 40–49, three in the age group < 30 years and only two were aged ≥ 60 years.

Twenty-one respondents (*n* = 21) were employed full-time in the special schools and only one (*n* = 1) was a part-time employee. One respondent, apart from being an employee at a school, was also a member of the District Based Support Team (DBST). The number of years they had been working at their current workplace ranged from 1.5 to 32 years with a mean of 10.57 ± 9.03 years.

The majority (*n* = 16) had a bachelor’s degree followed by four (*n* = 4) with both bachelor’s and master’s degrees. The number of years that they had been practising as physiotherapists ranged from 6 to 38 years with a mean and standard deviation of 18.3 ± 9.8 years. Six reported having ≤ 10 years of working experience as physiotherapists. One did not mention his or her work experience.

Twenty-one (95%) indicated that they knew about the SA *Education White Paper 6* (EWP6) *on Special Needs Education*. Of these, 19 (89%) responded to the question about ‘knowledge on the role of physiotherapists’ in ‘Inclusive Education’. The respondents were also asked to elaborate on the role of the physiotherapist in Inclusive Education and their statements were thematically analysed. The themes and sub-themes of the role of physiotherapists in ‘Inclusive Education’ are shown in Table 2.

**TABLE 2:** Role of Physiotherapist in Inclusive Education (section of questionnaire where respondents were asked to elaborate on their role in special schools).

Themes	Sub-themes
1) Analysis of learner needs	1.1 Assess the needs of the learners
	1.2 Identify aims for therapy to be addressed
	1.3 Meet a learner’s needs
2) Render physiotherapy services within the school environment	2.1 Render physiotherapy services in school
	2.2 Render physiotherapy services in the community
	2.3 Perform therapy in the classroom set-up
	2.4 Formulate treatment plans based on assessment, implementing treatment plans
	2.5 Continuation of rehabilitation and supporting education during transitory periods
3) Enable learners to access the curriculum	3.1 Assist the learners to access the curriculum by assisting educators in optimising learners with barriers to access to education
	3.2 Play a leading role in preparing a child with special needs to access formal education in the classroom; be it in a special school or in an inclusive setting in a full-service school, or a mainstream school
4) Empowerment of others	4.1 Share ‘best practice’ strategies with other therapists
	4.2 Offer in-service training and develop programmes, for example, special gross motor activity sessions that can be implemented in schools
	4.3 Provide support and advice to staff in the learners’ own school setting and where possible to give advice to support staff and learners of other schools and children in the community who may not yet be in the school setting
5) Collaborative practices	5.1 Establish networks for a multi-disciplinary approach (communicate with professionals from other disciplines to foster team functioning)
	5.2 Working towards a collaborative approach and collaborative goal to optimise the learner’s learning and function within the learning environment
	5.3 Be integrated into a District Based Support Team to provide specialised support to full-service schools or individually as needed in the district
	5.4 Be part of a multidisciplinary team to assist (the team) in the assessment of learners with barriers to learning and development (including disability) and to suggest interventions to address these barriers, including any support mechanism for the learners to achieve their maximum potential
	5.5 Communicate with parents with regard to their child’s home programme and give advice
	5.6 Give advice to parents and teachers on how to assist the child to cope, develop and learn optimally in his environment and school setting
6) Support other schools	6.1 Provide support to normal (mainstream) schools so that learners with special needs can attend mainstream (full-service/mainstream) schools.
7) Facilitate access to local resources available	7.1 Ensure the physical well-being and safety of learners with physical impairments while at school, for example, by ensuring safe mobility/specialised seating
	7.2 Identification of barriers and addressing any physical impairments that may hamper full participation of a learner
	7.3 Assist the learners in accessing their local school or support them in a full-service school

**TABLE 3:** Continuing Education and support from stakeholders involved in education (%).

Variable	Rating	*N*	%	Province 1		Province 2		Province 3		Province 4		Province 5	
				*n*	%	*n*	%	*n*	%	*n*	%	*n*	%
Continuing educational efforts	Poor	2	9	1	16.7	-	-	-	-	-	-	1	12.5
	Fair	3	14	1	19.7	-	-	-	-	-	-	2	25.0
	Good	10	46	3	50.0	-	-	3	70.0	1	100	3	37.5
	Very good	7	32	1	16.7	3	100.0	1	25.0	-	-	2	25.0
Support received regarding policies and processes	Poor	8	36	2	33.3	2	66.7	-	-	1	100	3	37.5
	Fair	7	32	2	33.3	-	-	1	25.0	-	-	4	50.0
	Good	6	27	2	33.3	1	33.3	2	50.0	-	-	1	12.5
	Very good	1	5	-	-	-	-	1	25.0	-	-	-	-

The respondents were asked to indicate the services they were currently providing to learners with special needs. Twenty-one (*n* = 21) responded to the question, and most of them (15; *n* = 15) provided an integrated service to learners which entailed a combination of consultative and direct therapeutic services. Six (*n* = 6) physiotherapists provided only direct services (one on one interaction) to learners. In the study by Pillay (2011), she found that many of the physiotherapists were experiencing difficulties in making the shift from direct to indirect service because of not having been provided with the necessary support from superiors, resources, and training to facilitate the transition to inclusive education practices.

In Table 4, the details of the examples of support or inadequate or poor support are listed.

**TABLE 4:** Categorisation of support received by respondents.

Adequate Support	Inadequate or Lack of Support
‘Good guidance from HOD. Flexibility of HOD to implement new programs and good opportunities to change and adapt programs’ (Respondent 4).	‘Our direct supervisor is not a principal. She [*the direct supervisor*] is the inclusive Education Coordinator and she does not give us a lot of guidance and support’ (Respondent 1).
‘I received adequate support from the other therapists and the principal’ (Respondent 9).	‘Not aware of any specific policies regarding physiotherapists. Did not receive any support or guidance on how to run a physiotherapy practice [*department*] in a school setting. As we settled in and started to know the learners and how the school operated, we implemented our therapeutic interventions and adapted continuously as we realized what worked and what didn’t. I had to take on many educational activities e.g., break time and afternoon supervision, driving school bus, translation work etc. to fit into the school environment while still maintaining our therapy programmes’ (Respondent 2).
‘We get good support from our school. We had difficulty accessing equipment/appliances for the DoH [*Department of Health*]. It’s a challenge. So, we source from NGOs/private donors etc’. (Respondent 8).	‘There is no guidance in how to run physiotherapy practice [*Department*] in a school set-up. We do what we think would work best and grow and adapt our approaches, therapy session, rosters etc. as needed’ (Respondent 3).
‘The school I work at is well resourced with a very supportive principal. The physios [*physiotherapists*] are represented in all major decision making meetings held at school’ (Respondent 12).	‘Most of the focus [*in our school*] is on education and therapists are excluded from the decisions [*physiotherapist not consulted to share their views*] by the DoE. … To date we do not have job descriptions and are understaffed. Therefore, policy and processes relating to therapy is scarce and documentation is not available’ (Respondent 7).
‘Financial and leave support was granted from the school as well as from the Department to attend professional training and workshops on a regular basis on various topics and aspects. We are always well informed about policies, process and changes taking place. The previous regime also had inspectors for physio departments laying a sound foundation’ (Respondent 16).	‘The DST is non-existent in my district. The SNES personnel are very scarce and offer no support. The procurement of assistive devices through DoE is limited and budget related. Poor school – limited budget’ (Respondent 13).
-	‘Not much input via Education department’ (Respondent 14).
-	‘We have had some training from the DoE but it isn’t frequent [*not regular continuous professional developmental activities*]. Also, to date we do not have an official job description’ (Respondent 15).
-	‘We need better support from the Education Department, with regards to the policies. We need people who have experience in therapy and education who can guide and direct us!’ (Respondent 18).
-	‘There is absolutely no support. Principal and most of the school management team have no idea what the profession is and what our roles are even after numerous attempts to promote Physiotherapy and even other therapies in the multidisciplinary team’ (Respondent 19).
-	‘We feel that we do not get enough support from WCED [*Western Cape Education Department*] (our employer) with regards to specific roles (PT [*physiotherapy*] vs. OT [*occupational therapy*] vs. SLT [*speech and language therapy*]). We also do not receive specific support with regards to policies and processes’ (Respondent 20).
-	‘While there are policies in place, there is no discussion as to how the policy was decided on, or how implementation will be effected. [*strategies to roll-out EWP6*] Not even on what or how the policy affects treatments’ (Respondent 21).
-	‘Support from my colleagues are good, but support from DoE not so good. Also very little training on school set-up in university or postgrad courses’ (Respondent 11).

HOD, head of department; DoH, Department of Health; DoE, Department of Education; DST, District Based Support Team; SNES, Special Needs Education Services; WCED, Western Cape Education Department.

The support from school management and DoBE was confirmed to be an area of concern amongst the respondents. Pillay (2011) found that the physiotherapists in her study required assistance with the transition from providing mainly direct support to children in special schools, to also providing indirect support to children in an inclusive education setting without the necessary support, resources and training to facilitate the transition.

In our study, we established how the learners were referred for physiotherapy. The respondents could give more than one response (Table 5).

**TABLE 5:** Referral pathway of learners requiring physiotherapy services.

Referred by	Number
Parents	3
Teachers	7
Principal	3
District Based Support Team	12
Screening by physiotherapists	5

Just more than half, 12 (*n* = 12) indicated that the learners who required physiotherapy services were referred by the DBST followed by referrals from teachers at seven (*n* = 7).

On the question to determine the extent to which undergraduate training prepared the respondents to work in a school setting, 19 (*n* = 19) indicated that their undergraduate training did not adequately prepare them for working in this kind of setting.

To follow-up on the roles of the physiotherapist in ‘Inclusive Education’ and the competencies identified by Effgen et al. (2007), the respondents were then asked to do a self-rating of their knowledge and skills. Their responses are summarised in Tables 6 and 7 below.

**TABLE 6:** Knowledge on issues relating to working in educational setting for learners with special needs.

Item	Poor		Fair		Good		Very good	
	*n*	%	*n*	%	*n*	%	*n*	%
Knowledge of the structure, goals, and responsibilities of the public educational system to meet the educational needs of the children with special needs. (Q17)	1	5.0	6	27.0	13	59.0	2	9.0
Awareness of educational standards, curricula, and general and special education teaching strategies, to enable you to design and implement effective support and service. (Q18)	3	13.6	8	31.8	10	40.9	3	13.6
Familiar with community resources and school extracurricular programmes is necessary to promote children’s full participation in age appropriate social and physical activities. (Q19)	3	13.6	5	22.7	11	50.0	3	13.6
Knowledge of educational system and its critical components, including the government regulations, ethical/legal responsibilities that apply to physiotherapists in the educational setting for children with special needs. (Q20)	1	5.0	10	50.0	8	40.0	1	5.0
Knowledge of disabling conditions that occur before and after birth, as well as the effects on learners as related to their educational performance. (Q21)	1	5.0	2	9.1	11	50.0	8	36.4
Knowledge of major theories, treatment procedures, and research relevant to providing physiotherapy services for children (infants through to age 22) with disabilities. (Q22)	-	-	3		12	54.5	7	31.8
Knowledge of the structure, goals, and responsibilities of the public educational system to meet the educational needs of the children with special needs you serve. (Q23)	-	-	11	50.0	10	45.5	1	5.0

**TABLE 7:** Respondents’ opinion on their skills for working in an educational setting.

Item	Poor		Fair		Good		Very good	
	*n*	%	*n*	%	*n*	%	*n*	%
Ability to assess the functional performance of learners with disabilities within the school environment. This would include identifying, comparing, selecting, and administering appropriate evaluations.	-	-	2	9.1	12	54.5	8	36.4
Ability to interpret assessment results appropriately and use the results to develop therapeutic intervention plans and classroom strategies appropriate to the educational goals for the student. This could include adaptive equipment, assistive technology devices, and external professional consultation.	-	-	-	-	14	63.6	8	36.4
Ability to adapt the child’s environments to facilitate learner access to and participation in learner activities.	-	-	-	-	15	68.2	7	31.8
Ability to use various types and methods of service provision in intervention including direct, individual, group, integrated, consultative, monitoring, and collaborative approaches.	-	-	2	9.1	16	72.7	4	18.2
Ability to embed therapy interventions into the context of learner activities and routines and as appropriate use activity-based and play-based approaches that optimise learning opportunities within natural contexts.	1	5.0	1	5.0	17	77.3	3	13.6
Ability to be reflective, critically evaluate your intervention approaches, and use evidence-based interventions.	1	5.0	5	22.7	14	63.6	2	9.1
Ability to communicate effectively (both orally and in writing) with educational personnel, parents, local and government agencies, and the community at large.	1	5.0	1	5.0	14	63.6	6	27.3
Ability to form partnerships amongst family members, service providers, and the community to provide coordinated care.	2	9.1	3	13.6	12	54.5	5	22.7
Ability to function as consultants to school personnel and families to promote the inclusion of the learner in the educational experience.	-	-	3	13.6	14	63.6	5	22.7
Ability to coordinate services across the home, school, medical, and community settings.	-	-	8	36.4	11	50.0	3	13.6
Ability to supervise personnel and professional staff to ensure quality care.	-	-	3	13.6	18	81.8	1	5
Ability to engage in consensus decision-making as part of the Individualized Education Plan (IEP) process in order to write IEP goals and, if appropriate, objectives or benchmarks.	1	5.0	6	27.3	11	50.0	4	18.2
Ability to plan and implement intervention strategies using a continuum of service delivery approaches in accordance with learner needs in the least restrictive educational environment.	-	-	8	36.4	13	59.1	1	5.0
Ability to evaluate, modify, and document the effectiveness of physiotherapy intervention as it relates to the learner’s educational programme.	-	-	6	27.3	13	59.1	3	13.6
Ability to facilitate transition between agencies, programmes, and professionals as service provision changes (early intervention to preschool, preschool to school age, and school to work).	4	18.2	10	45.5	6	27.3	2	9.1

From Table 6, it becomes clear that between 32% and 55% of the respondents’ knowledge on matters regarding the goals and aims of the educational system; its standards, curricula, general as well as special education teaching strategies, to enable physiotherapists to design and implement effective support service to the learners varied between ‘poor to fair’, and government regulations, ethical and/or legal responsibilities that apply to physiotherapists, varied between poor and fair. However, around 85% reported that their knowledge on clinical matters was good to very good.

Ninety-five percent (95%) indicated that they knew about the Education White Paper 6, policy as Rapport and Effgen (2004) mentioned the importance of a school-based paediatric physiotherapist being knowledgeable about civil and educational laws.

*Education White Paper 6 on Special Needs Education* was implemented in the last two decades and more attention still needs to be given to continuing professional development (CPD) of physiotherapists in SA. The CPD will equip physiotherapists to better understand their role in the educational system and teaching strategies, in order to enable them to design and implement effective support and intervention services for learners.

The respondents’ opinions on their clinical skills (Table 7) varied between good and very good. However when correlated with the findings presented in Table 6, the rating of the skills that related to the integration of the clinical physiotherapy goal setting, with formulating individualised education programmes (IEPs), knowledge and understanding of the educational system, planning partnerships with the family, other service providers, and community initiatives, some respondents rated their skills poor or fair.

Rapport and Effgen (2004) acknowledged that skills such as clinical, administrative, and technical skills required of school-based physiotherapy services are not part of a professional entry-level physiotherapy programme.

However, competencies are more complex than a ‘skill’ and include reflection, problem-solving ability and behaviour, as well as knowledge. In our study, we regarded it as important to establish the correlation between knowledge and skills and the results are presented in Table 8.

**TABLE 8:** Correlation between physiotherapists’ knowledge and skills.

Skills Questions	Q17	Q18	Q19	Q20	Q21	Q22	Q23
Ability to assess the functional performance of learners with disabilities within the school environment.	0.259	0.250	0.229	0.443	0.444*	0.631*	0.333
Ability to interpret assessment results appropriately and use the results to develop therapeutic intervention plans and classroom strategies appropriate to the educational goals for the student.	0.255	−0.014	0.153	0.129	0.424	0.463*	0.133
Ability to adapt the child’s environments to facilitate learner’s access to and participation in learner activities.	0.402	0.394	0.367	0.466*	0.483*	0.638*	0.386
Ability to use various types and methods of service provision in intervention including direct, individual, group, integrated, consultative, monitoring, and collaborative approaches.	0.059	0.063	−0.157	0.029	0.206	0.373	0.238
Ability to embed therapy interventions into the context of learner activities and routines and as appropriate use activity-based and play-based approaches that optimise learning opportunities within natural contexts.	0.452	0.339	0.304	0.586*	0.275	0.605*	0.558*
Ability to be reflective, critically evaluate your intervention approaches, and use evidence-based interventions.	0.041	−0.094	0.321	0.126	0.396	0.279	0.242
Ability to communicate effectively (both orally and in writing) with educational personnel, parents, local and government agencies, and the community at large.	0.311	0.000	0.218	0.298	0.234	0.598*	−0.059
Ability to form partnerships amongst family members, service providers, and the community to provide coordinated care.	0.482*	0.185	0.689*	0.533*	0.302	0.579*	0.193
Ability to function as consultants to school personnel and families to promote the inclusion of the learner in the educational experience.	0.373	0.338	0.295	0.573*	0.290	0.664*	−0.032
Ability to coordinate services across the home, school, medical, and community settings.	0.308	0.140	0.374	0.221	0.339	0.353	0.386
Ability to supervise personnel and professional learner to ensure quality care.	−0.073	−0.078	−0.213	−0.037	0.102	0.041	0.141
Ability to engage in consensus decision-making as part of the Individualized Education Plan (IEP) process in order to write IEP goals and, if appropriate, objectives or benchmarks.	0.087	0.256	0.034	−0.029	0.067	0.223	−0.072
Ability to plan and implement intervention strategies using a continuum of service delivery approaches in accordance with learner needs in the least restrictive educational environment.	−0.048	0.165	0.103	−0.156	−0.001	−0.045	−0.069
Ability to evaluate, modify, and document the effectiveness of physiotherapy intervention as it relates to the learner’s educational programme.	0.181	0.140	0.374	0.324	0.339	0.465	0.216
Ability to facilitate transition between agencies, programmes, and professionals as service provision changes (early intervention to preschool, preschool to school age, and school to work).	0.121	0.167	0.373	0.176	0.508	0.349	0.516*

* , *p* < 0.05.

The respondents’ knowledge of the structure, goals, and responsibilities of the public educational system to meet the educational needs of children was positively associated with all the skill questions except with the ability to supervise personnel and professional learners (university students) in order to ensure quality care as well as the ability to plan and implement intervention strategies using a continuum of service delivery approaches in accordance with learner needs in the least restrictive educational environment. However, the results were not statistically significant (*r* = –0.073; *p* < 0.05). Similarly, the knowledge of the structure, goals, and responsibilities of the public educational system to meet the educational needs of children, was significantly associated with the ability to form partnerships amongst family members, service providers, and the community to provide coordinated care (*r* = 0.482; *p* < 0.05). This finding makes sense as departmental leaders (managers) are primarily responsible for the administrative duties within the physiotherapy department and would not spend as much time being directly involved with the supervision of personnel and physiotherapy students and implementation of intervention strategies. However, the managers would be more skilled in forming partnerships amongst family members, service providers, and the community to provide coordinated care. This finding is similar to McGowan, Martin and Stokes (2016) who compared Canadian and Irish physiotherapists’ views on leadership and found that effective interactions with patients and their families, as well as people working within the health system were important in good leadership.

## Discussion

### Knowledge of EWP6 and the role of physiotherapy in inclusive education

Twenty-one (95.5%) respondents indicated that they knew about the *Education White Paper 6* (EWP6) *on Special Needs Education*. Of these, 19 indicated that they knew about the role of the physiotherapist in inclusive education. The themes and sub-themes identified in their responses (Table 2) supported their answer. However, they indicated that they had a need to know more on the implementation of this policy and how it should be rolled-out.

From Table 3 and Table 4, it is clear that the support that physiotherapists received from the DoBE varied between the provinces. In some provinces it was adequate and in some others it was inadequate. The national DoBE should focus on developing functional DBST’s to support physiotherapists and facilitate the implementation of EWP6 at a district level. We found that the support by DoBE for physiotherapists, varied (was adequate in some schools/provinces and inadequate in others). Furthermore, it is now understood from our study that although generic competencies for physiotherapy are also relevant for physiotherapy practice in an educational setting, the respondents needed and displayed competencies that were specific and went beyond the generic competencies.

Correlations were found to exist between knowledge and skills in survey respondents. However, the knowledge and skills required were related to the physiotherapists’ roles and responsibilities they had within the departments. For example, physiotherapy managers had more knowledge about the structure of public education and thus they are more skilled in forming partnerships with service providers to provide co-ordinated care. Thus, the competencies required and displayed were determined to a large extent by the responsibilities that the physiotherapist had in that department.

Effgen et al. (2007) emphasised that physiotherapists’ professional development in school-based practice is fourfold and similar to professional development for physiotherapists in early intervention. Firstly, physiotherapists must develop competencies in the broad body of knowledge and skills related to paediatric physiotherapy. Secondly, they must have knowledge of the professional, government policies, government rules and regulations as well as guidelines to practice in schools. Thirdly, physiotherapists need to acquire the global knowledge and skills to work in a school setting. Fourthly, they need to be mentored during on-the-job training and maintain a dedication to life-long learning to promote state-of-the-art, evidence-based practice. The training of physiotherapists in school-based services should be discipline specific as well as interdisciplinary to reflect the collaboration required in schools (DoBE, school principals, and other stakeholders).

## Conclusion

Our study was conducted to determine the range of competencies that are needed for physiotherapists to render a service for children with special needs in an educational environment.

In addition to the generic competencies required of physiotherapists in SA, the physiotherapists are required to have in-depth knowledge of *Education White Paper 6* and the processes of its implementation.

Although 78% of the physiotherapists indicated that they have good to very good support in terms of CPD, 68% reported that their support in terms of policy implementation and processes in special schools, are fair to poor.

Whereas their fair to poor support in terms of CPD activities is limited to mainly two provinces, the lack of support in terms of policy implementation and processes, is spread over all five provinces whose physiotherapists completed the questionnaire.

The physiotherapists’ rating of their knowledge on the educational system and its critical components, including government regulations, ethical and/or legal responsibilities that apply to physiotherapists in the educational setting for children with special needs, and are rated between fair and poor by 30% – 55% of the respondents. This tendency is also reflected in the physiotherapists’ rating of their skills. The lack of knowledge in the educational policies and procedures, seems to limit the majority of the physiotherapists’ ability to integrate their clinical skills with the educational settings. Lack of integration of professional knowledge influences the quality of service rendered in the schools for children with special needs.

It is clear that the implementation of EWP6 is ‘a process’ and not ‘an event’. The SA national DoBE and the management of special schools should continuously review their services and processes, and need to work collaboratively with physiotherapists to eventually enable learners with special needs to access their school curriculum in a manner that is fair and just, alongside their peers, to the best of their ability.
